# Analysis of Access to Prescription Data Management Programs Data for Research

**DOI:** 10.1001/jamanetworkopen.2022.18094

**Published:** 2022-06-22

**Authors:** Vivian A. Lee, Wilson M. Compton, Jonathan D. Pollock

**Affiliations:** 1 National Institute on Drug Abuse, National Institutes of Health, Bethesda, Maryland; 2 Genetics, Epigenetics, and Developmental Neuroscience Branch, Division of Neuroscience and Behavior, National Institute on Drug Abuse, National Institutes of Health, Rockville, Maryland

## Abstract

This cross-sectional study examines the availability of prescription drug monitoring program data for research and whether those data can be linked to other data.

## Introduction

In response to the ongoing opioid crisis,^1,2,3^ 50 states and 4 territories (Puerto Rico, District of Columbia, Northern Mariana Islands, and Guam) enacted prescription drug monitoring programs (PDMPs) to reduce risky prescribing of opioids by health care practitioners, decrease misuse of controlled substances, and decrease overdose deaths. Although health care practitioners can access PDMP data for clinical purposes through online information-sharing hubs such as PMPGateway and RxCheck, policies for access to PDMP data and linking of PDMP data to other data for research purposes are unclear for many jurisdictions. PDMP data have been used to evaluate high-risk use and prescribing practices, to improve data quality, to determine health outcomes, and to predict opioid overdose.^4,5,6^ Linking of PDMP data to electronic health records and other data has the potential for repurposing medications to treat pain and addiction, for identifying comorbid health conditions, for identifying genetic variants associated with addiction, and for determining how to best target resources and interventions to address opioid misuse. The objective of the current cross-sectional study is to determine the availability of PDMP data for research and whether PDMP data can be linked to other data.

## Methods

PDMP managers from 54 jurisdictions were contacted by email from December 2020 to October 2021, with data analysis completed by December 2021. All PMDP managers except 1 responded to the 4 questions by email about the type of PDMP data permitted to be shared, whether PDMP data can be linked to other data sources for research purposes, whether linking of PDMP data requires a secure mechanism such as an honest broker, and whether institutional review board (IRB) approval is required to access PDMP data and to link to other data. Our analysis about access and linking is based on response to questions and written policy statements received from PDMP managers regarding data access. No IRB approval or consent was required because questions about policy are nonhuman participant research according to the National Institutes of Health IRB (see the eAppendix in the Supplement for question wording). Responses to the questions were entered into an Excel spreadsheet version 2108 (Microsoft). The Someka Heat Map of the US was used to show the map of PDMP data access policies in different jurisdictions.

## Results

**Figure 1.**
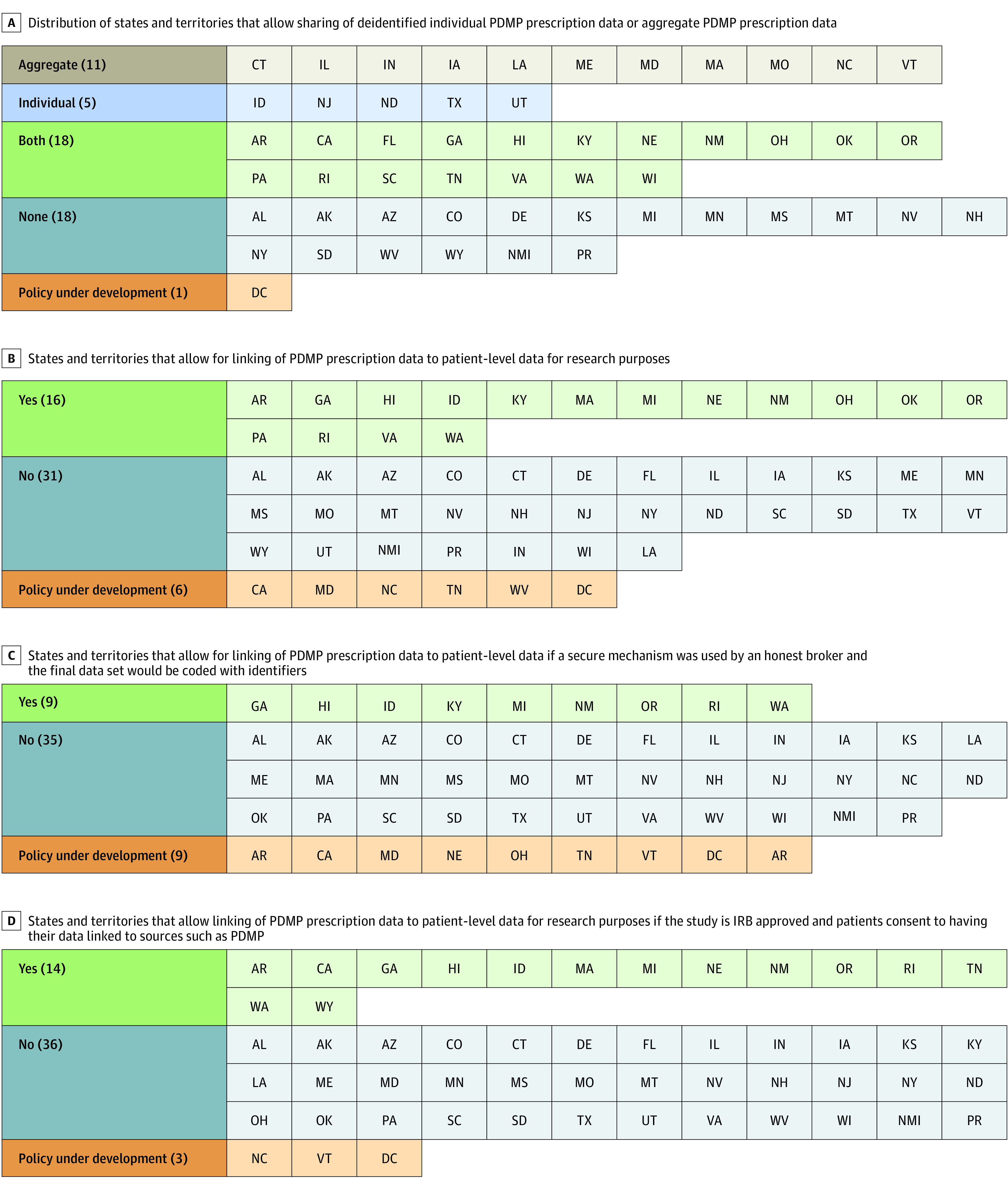
State Prescription Drug Monitoring Program (PDMP) Policy Distributions A, Distribution of states and territories that allow sharing of deidentified individual PDMP prescription data or aggregate PDMP prescription data. B, States and territories that allow for linking of PDMP prescription data to patient-level data for research purposes. C, States and territories that allow for linking of PDMP prescription data to patient-level data if a secure mechanism was used by an honest broker and the final data set would be coded with identifiers. D, States and territories that allow linking of PDMP purposes if the study is institutional review board approved and patients consent to having their data linked to sources such as PDMP. NMI indicates Northern Mariana Islands.

**Figure 2.**
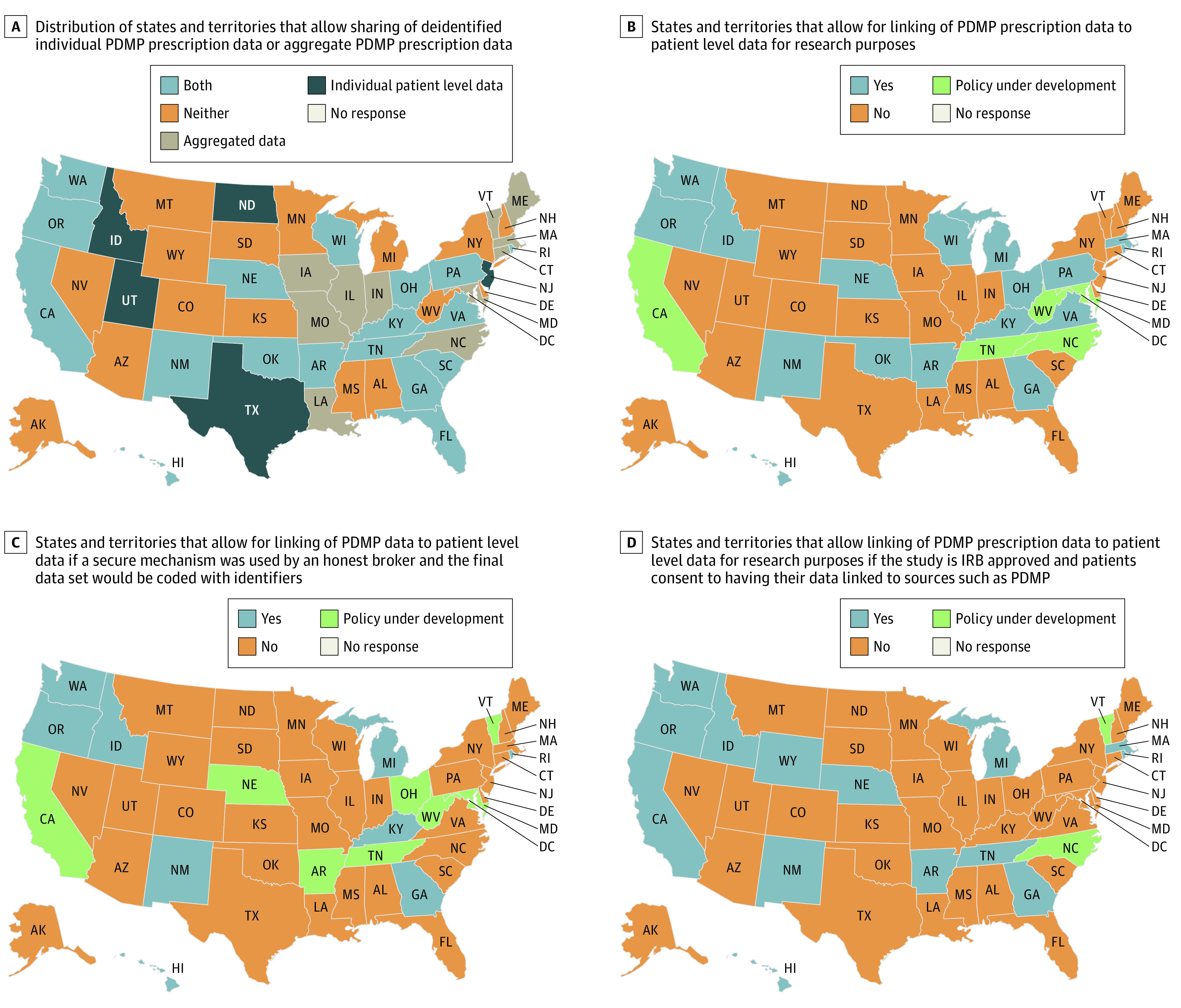
Map of Prescription Drug Monitoring Program (PDMP) Data Access Policies for Research The figure depicts the results of the PDMP query in the form of heat maps to visualize patterns in results for different regions. The heat maps illustrate the difficulties in obtaining complete patient histories of scheduled prescription use in states where PDMP access is granted, especially among patients who can cross state boundaries to fill scheduled prescriptions. The figure was generated using the heat map template by Someka Excel Solutions (https://www.someka.net/excel-template/usa-heat-map-generator/).

The results of the survey from 53 jurisdictions showed inconsistent policies for accessing PDMP data for research purposes. Only 23 states permit access to deidentified PDMP data for research purposes, 16 states permit the linking of PDMP data with patient-level data for research purposes, and 9 states permit the use of an honest broker system to link deidentified PDMP data to electronic health records (Figure 1 and Figure 2). In no state except Hawaii do states permit interstate data sharing for research purposes, which results in an incomplete patient history of the use of controlled substance use.

## Discussion

Although a limitation of the current cross-sectional study is a possible misunderstanding of the questions by PDMP managers, the results demonstrate that there are large differences among jurisdictions in accessing PDMP data and linking PDMP data to other data sources for research. These differences, the inability to access PDMP data across jurisdictional boundaries, and limitations on linking PDMP data impede the utility of PDMP data for research. Policies that prevent access to data across jurisdictions produce incomplete patient histories of controlled substance use and may result in biased studies. Creating uniform policies across jurisdictions, easing access to PDMP data, and linking PDMP data to other data sources will greatly enhance opportunities for developing new interventions, repurposing medications for opioid addiction, conducting research on the genetics of opioid addiction, and improving PDMP data quality. Uniform state and federal laws and policies should be established to enable research using the PDMP data and data hubs that protect privacy through a single access point. As the US overdose epidemic persists^2^, it is imperative that action be taken to reform current PDMP policies.

## Notes

Supplement. **eAppendix.** Supplemental Methods
