# Supplementary material for: Analysis of Access to Prescription Data Management Programs Data for Research
Source: JAMA Netw Open. 2022 Jun 22;5(6):e2218094. doi: 10.1001/jamanetworkopen.2022.18094 (PMC9218843; doi:10.1001/jamanetworkopen.2022.18094)
Supplement: Supplement. — eAppendix. Supplemental Methods [file jamanetwopen-e2218094-s001.pdf]

Supplemental Online Content

Lee VA, Compton WM, Pollock JD. Analysis of access to prescription data management programs data for research. *JAMA Netw Open*. 2022;5(6):e2218094. doi:10.1001/jamanetworkopen.2022.18094

eAppendix. Supplemental Methods

This supplemental material has been provided by the authors to give readers additional information about their work.

© 2022 Lee VA et al. *JAMA Network Open.*

eAppendix. Supplemental Methods

The wording of the four questions sent by email to PDMP manager: 1) Does your state/territory allow sharing of de-identified individual PDMP prescription data or aggregate PDMP prescription data? 2) Does your state/territory allow linking of PDMP prescription data to patient level data for research purposes? 3)Does your state/territory allow linking of PDMP data to patient level data if a secure mechanism was used by an honest broker and the final dataset would be coded with identifiers? 4) Does your state/territory allow linking of PDMP prescription level data for research purposes if the study is IRB approved and patients consent to having their data linked to sources such as PDMP? PDMP administrators were asked to provide hyperlinks to written policy statements and laws regarding access by researchers, health providers, and law enforcement. Following written replies clarification was followed up with emails and phone calls.

© 2022 Lee VA et al. *JAMA Network Open.*
